# Metformin attenuates cartilage degeneration in an experimental osteoarthritis model by regulating AMPK/mTOR

**DOI:** 10.18632/aging.102635

**Published:** 2020-01-16

**Authors:** Xiaofeng Feng, Jianying Pan, Junyan Li, Chun Zeng, Weizhong Qi, Yan Shao, Xin Liu, Liangliang Liu, Guozhi Xiao, Haiyan Zhang, Xiaochun Bai, Daozhang Cai

**Affiliations:** 1 Department of Orthopedics, Academy of Orthopedics Guangdong Province, Orthopedic Hospital of Guangdong Province, Guangdong Provincial Key Laboratory of Bone and Joint Degenerative Diseases, The Third Affiliated Hospital of Southern Medical University, Guangzhou 510280, China; 2 Department of Biology and Shenzhen Key Laboratory of Cell Microenvironment, South University of Science and Technology of China, Shenzhen 518055, Guangdong, China; 3 Key Laboratory of Mental Health of the Ministry of Education, Department of Cell Biology, School of Basic Medical Sciences, Southern Medical University, Guangzhou 510515, Guangdong, China; 4 Guangzhou Regenerative Medicine and Health Guangdong Laboratory, Guangzhou 510005, China

**Keywords:** metformin, cartilage injury, osteoarthritis, AMPK/mTOR

## Abstract

Background: It is generally thought that the occurrence and progression of osteoarthritis (OA) results from multiple causes, including degradation and destruction of the cartilage matrix and aging of chondrocytes. Metformin is a first-line drug for the treatment of diabetes, and has great potential for the treatment of other disorders. However, the role of metformin in OA is unknown.

Results: Metformin displayed a protective effect against OA. There were lower OARSI scores and fewer MMP-13-positive cells in DMM mice and cartilage explants after treatment with metformin. In addition, metformin treatment decreased p16^INK4a^ levels in OA chondrocytes, and enhanced polarization of AMPK and inhibition of mTORC1 in OA mice and chondrocytes in a dose-dependent manner.

Conclusions: Metformin effectively alleviated cartilage degradation and aging through regulation of the AMPK/mTOR signaling pathways, suggesting that it could be an effective treatment for OA.

Methods: The effects of metformin on cartilage degradation and chondrocyte aging was determined in a destabilization of the medial meniscus (DMM)-induced OA mouse model and in IL-1β-treated mouse chondrocytes and cartilage explants. Articular cartilage degeneration was graded using the Osteoarthritis Research Society International (OARSI) criteria. Immunostaining, RT-PCR, and western blot analyses were conducted to detect the relative expressions of protein and RNA.

## INTRODUCTION

Osteoarthritis (OA) is a chronic, multicausal, and progressive joint disease that is most common in middle-aged and older patients [1–2]. The pathological symptoms of OA involve articular cartilage degeneration and destruction, subchondral bone sclerosis, reactive hyperplasia of the joint edge and subchondral bone, and the formation of osteophytes [3–6]. The pathological changes of OA are mainly characterized by degradation of the cartilage matrix and abnormal anabolism. As the disease progresses, the symptoms of OA are mainly characterized by joint pain, deformity, and deterioration of joint functions.

There are presently few drugs for the treatment of OA. Only anti-inflammatory and analgesic drugs are used to relieve OA symptoms [7–8]. The later periods of OA can only be treated with surgery, and it is still impossible to heal OA. Identifying an effective drug to heal OA is therefore urgently needed in the clinic.

Metformin is a first-line drug, used extensively for the treatment of diabetes [9–10]. In addition to its hypoglycemic effect, metformin also has anti-inflammatory, anti-tumor, and anti-aging activities, which indicate that metformin has great potential for the treatment of other disorders [11–13]. Research on metformin currently involves many aspects, but mainly focuses on its anti-inflammatory aspects, such as a possible treatment for rheumatoid arthritis, inhibition of tumor cells, anti-aging effects, and treatments for kidney disease, liver disease, and apoptosis metabolism [14–21]. Although there have been few studies on the role of metformin in OA, the number of related studies has increased in recent years. Most of the recent articles on treating OA with metformin focus on its anti-inflammatory and cartilage matrix protection effects [22–26]. However, the relevant mechanism is not yet clear, and there is still much room for exploration of related signal pathways. Adenylate-activated protein kinase (AMPK) is known as a “cell energy regulator,” [27–29] and plays a key role in the regulation of cellular energy homeostasis, and has short-term effects on energy metabolism regulation and long-term effects on gene metabolism regulation. Its negative regulation involves direct phosphorylation of a series of enzymes, or transcriptional regulation of metabolism by phosphorylated transcription factors, synergistic activators, and synergistic inhibitors. It also acts as an important regulator of aging by interacting with mTOR [30–31].

As previously mentioned, OA is a chronic and multi-causal progressive joint disease. Age is another important factor influencing the development of OA. Based on the characteristics of bone growth and development, the progress of OA is positively correlated with age [32]. At present, there have been few studies on the mechanism of chondrocyte aging in OA, but it is well-established that cartilage aging plays an important role in the progression of OA. Reports [33–34] on cell senescence in other fields have indicated that during cartilage aging, the accumulation of senescent cells leads to environmental changes in the extracellular matrix of the joint space. The accumulation of p16-positive chondrocytes can not only block cell proliferation, but also may have adverse effects on tissue regeneration, and it is possible that the secretion of matrix metalloproteins (MMPs) leads to degradation of the extracellular matrix [35–36]. The stability of the extracellular matrix is very important in protecting the integrity of the articular cartilage. An increase of p16^INK4a^ expression in senescent cells stimulates the secretion of MMPs, which directly enhances the degradation of collagen II. The degradation of type II collagen leads to the degradation of cartilage matrix. With chondrocyte aging, normal cell function is also reduced or even lost, ultimately affecting the synthesis of the cartilage matrix of cartilage cells, which damages the articular cartilage and promotes the occurrence and progression of OA.

Many studies have recently conducted in-depth research on the pathogenesis of OA, and have found specific markers and related signaling pathways, which are associated with the progression of OA, such as the AMPK/mTORC1 signaling pathways [37], which will have a great impact on the study of OA and the mechanism of action of drugs to treat this disorder. Studies [38] have reported that metformin acts as an activator of the AMPK signaling pathway, and efficiently activates the AMPK signaling pathway to affect bone metabolism. At present, there have been few reports on the effects of metformin on bone metabolism, and there have been even fewer studies involving OA. To bridge this gap, we have conducted a series of preliminary studies on the role of metformin in OA. The results of chondrocyte and mouse OA model experiments showed that metformin may inhibit the senescence of chondrocytes and the mTORC1 signaling pathway, and simultaneously activate the AMPK signaling pathway, thereby contributing to the treatment of OA.

## RESULTS

### Metformin attenuates cartilage degeneration in DMM mice and cartilage explants

**Figure 1:**
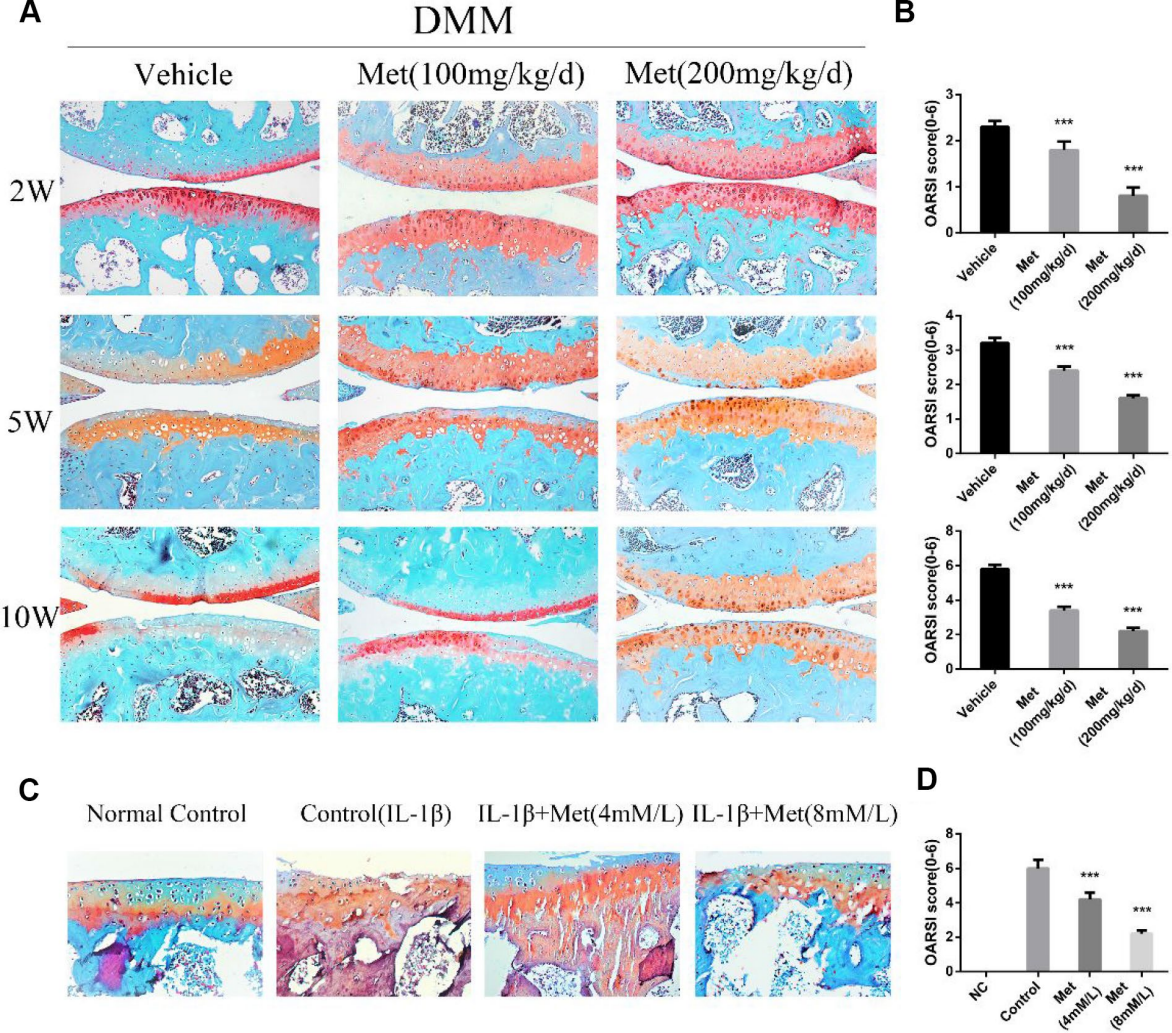
**Histological scoring of the destabilization of the medial meniscus (DMM), DMM + Metformin joints, and cartilage explants.** (**A**) Representative photographs of the joints of male mice at 2, 5, and 10 weeks after surgery. (**B**) Osteoarthritis Research Society International (OARSI) scoring of the joints of DMM, DMM + metformin male mice at 2, 5, and 10 weeks after surgery, as well as cartilage explants (**D**). (**C**) Representative Safranin O staining of paraffin section of mouse cartilage explants treated with IL-1β + metformin (4 mM and 8 mM) for 5 days. Sections were stained with Safranin O/Fast Green. Two weeks: n = 24; 5 weeks: n = 24; 10 weeks: n = 24. Cartilage explants: (n = 6 per condition). Values are expressed as the mean ± SEM. ^***^p < 0.001 compared with the control.

To determine the functional role of metformin in OA development, we administered metformin intragastrically in mice after DMM surgery, and the cartilage damage was assessed at 2, 5, and 10 weeks after surgery. Importantly, there was a protective OA phenotype in metformin-treated DMM mice. Safranin O staining demonstrated proteoglycan loss as early as 2 weeks after surgery, which developed into serious cartilage damage at 10 weeks. The cartilage degeneration was significantly reduced in mice treated with metformin, in a dose-dependent manner. These results were confirmed by the OARSI score (Figure 1A, 1B, Supplementary Figure 1A, 1B). We further confirmed these results in an organotypic model. In cartilage explants, metformin counteracted the IL-1β-induced loss of proteoglycans and cartilage degeneration in the superficial layer (Figure 1C). Overall, these results showed that metformin treatment protected articular cartilage during the progression of OA.

### Metformin reduces matrix-degrading enzyme production and promotes chondrocytes synthesis

**Figure 2:**
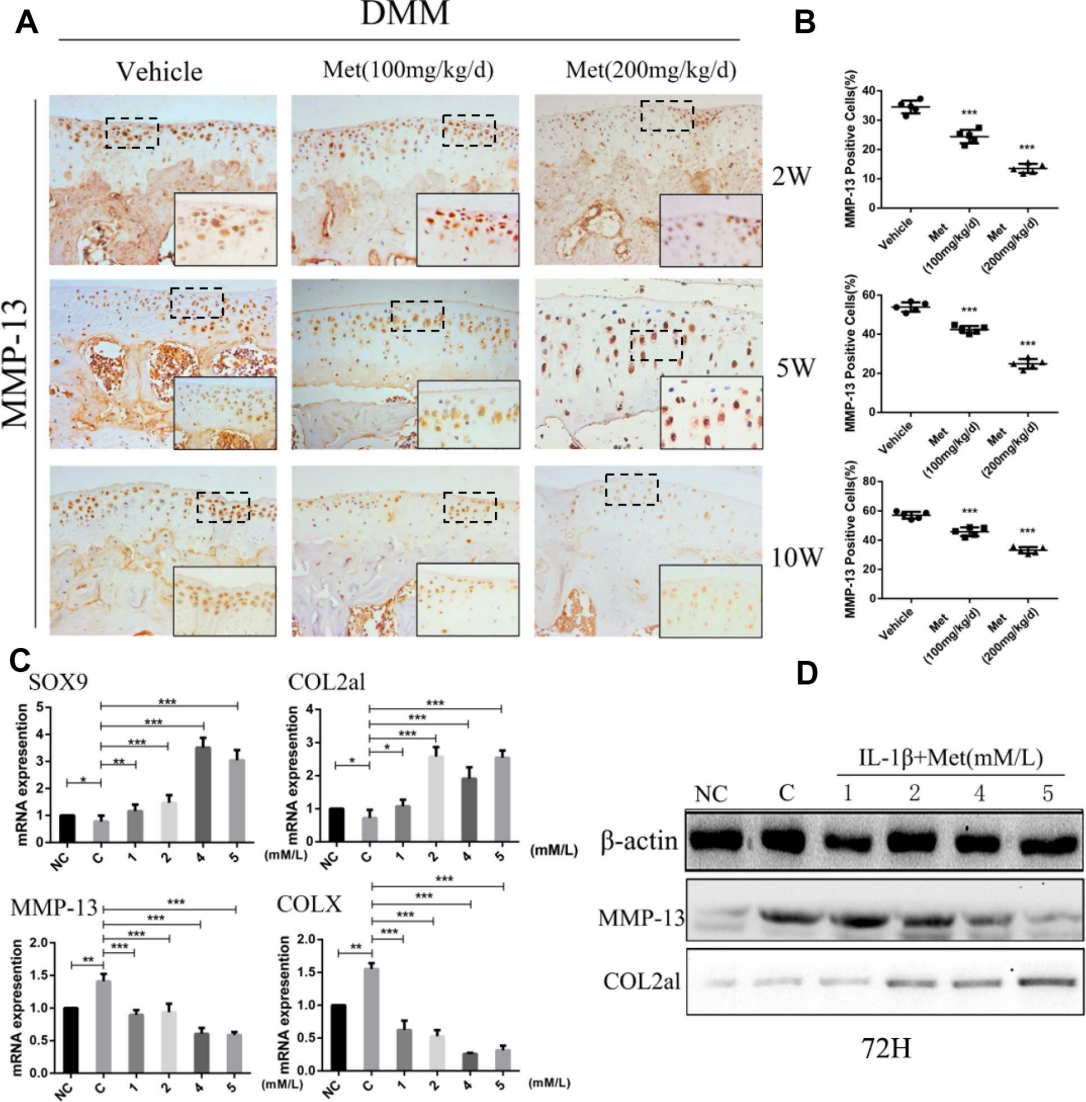
**Metformin reduces the degradation of cartilage matrix and stimulates its synthesis, while also affecting the formation of osteophytes.** (**A**) Immunohistochemical detection of MMP-13 in tibial cartilage at 2, 5, and 10 weeks after destabilization of the medial meniscus surgery. (**B**) Quantification of cells positively stained for matrix metalloproteinase-13 (MMP-13). (**C**) The mRNA expression levels of MMP-13, SOX9, COLX and COL2al, and (**D**) Western blot analyses of the protein expression levels of MMP-13 and COL2al. Primary chondrocytes were induced with IL-1β (10 ng/mL), and then co-cultured with metformin (1, 2, 4, and 5 mM) for 72 h. Two important cartilage matrix degrading proteins, MMP-13 and COLX, were decreased during administration, while other important cartilage matrix synthetic proteins, SOX9 and COL2al, were promoted during administration at 72 h. ^*^P < 0.05; ^**^P < 0.01; ^***^P < 0.001 between two groups.

We next analyzed the mechanism of metformin on cartilage matrix degradation and synthesis. Matrix metalloproteinase-13 (MMP-13) and matrix metalloproteinase-3 (MMP-3), which are widely studied in OA, play a crucial role in cartilage ECM degradation. Immunohistochemical analysis showed that the proportion of MMP-13-positive cells was significantly lower in metformin-treated mice compared with controls, especially at 10 weeks after DMM surgery (Figure 2A, 2B), and the MMP-3 had the same expression trend by immunofluorescence staining (Supplementary Figure 3A, 3B). To investigate the role of metformin during chondrogenesis, neonatal mouse primary chondrocytes were obtained and induced with IL-1β (10 ng/mL) for 24 h, and then co-cultured with different concentrations of metformin (1, 2, 4, and 5 mM) for 24 h, 48 h, and 72 h. Gene expression data indicated that as the concentration of metformin increased, the expressions of cartilage matrix degrading protein, such as COLX and MMP-13, were reduced, while the chondrogenic markers, SOX9 and Col2a1, were significantly increased (Figure 2C; Supplementary Figure 2A–2D). The expression of MMP13, MMP-3 and Col2a1 were confirmed by western blotting in primary chondrocytes (Figure 2D; Supplementary Figure 3C). Taken together, these results indicated that metformin decreased expressions of MMP-13, MMP-3 and ColX, and induced expressions of Col2a1 and SOX9, which led to reduced cartilage ECM degradation and promotion of chondrocytes synthesis.

### Metformin delays the aging of cartilage

**Figure 3:**
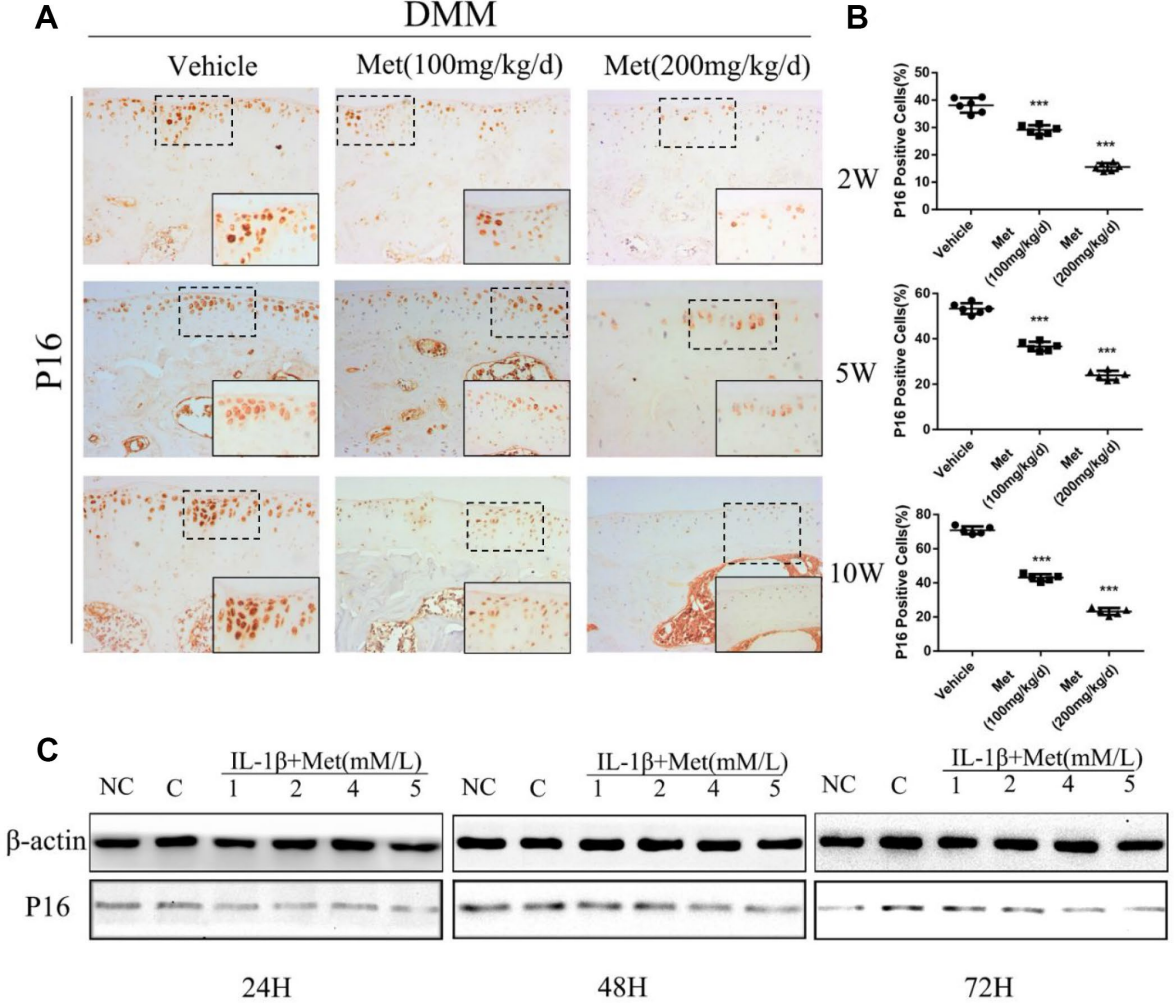
**Expression of the p16^INK4a^ protein in articular cartilage of the destabilization of the medial meniscus (DMM) model in mice.** (**A**) Immunohistochemical detection of p16^INK4a^ in tibial cartilage at 2, 5, and 10 weeks after DMM surgery. (**B**) Quantification of cells positively stained for p16^INK4a^. ^***^P < 0.001 between the two groups. And (**C**) the western blot analyses of the protein expression levels of p16^INK4a^ protein in primary chondrocytes which were induced with IL-1β (10 ng/mL) and co-cultured with metformin (1, 2, 4, and 5 mM) for 24 h 48 h and 72 h.

To determine whether metformin was involved in regulating chondrocyte aging in articular cartilage, we determined p16^INK4a^ expression and cellular senescence markers in chondrocytes of DMM mice. The percentage of p16^INK4a^-positive cells was significantly increased with the progression of articular cartilage degeneration. However, expression of p16^INK4a^ was maintained at lower expression by treatment with metformin (Figure 3A, 3B). To extend these findings, we transiently overexpressed metformin in mice primary chondrocytes. There was a significant increase in chondrocyte viability following metformin overexpression. Western blotting showed that p16^INK4a^ was significantly decreased in a dose-dependent manner in primary articular chondrocytes treated with metformin (Figure 3C). Together, the results showed that metformin treatment decreased cartilage aging both *in vivo* and *in vitro*.

### AMPK/mTORC1 signaling is responsible for the effect of metformin in OA

**Figure 4:**
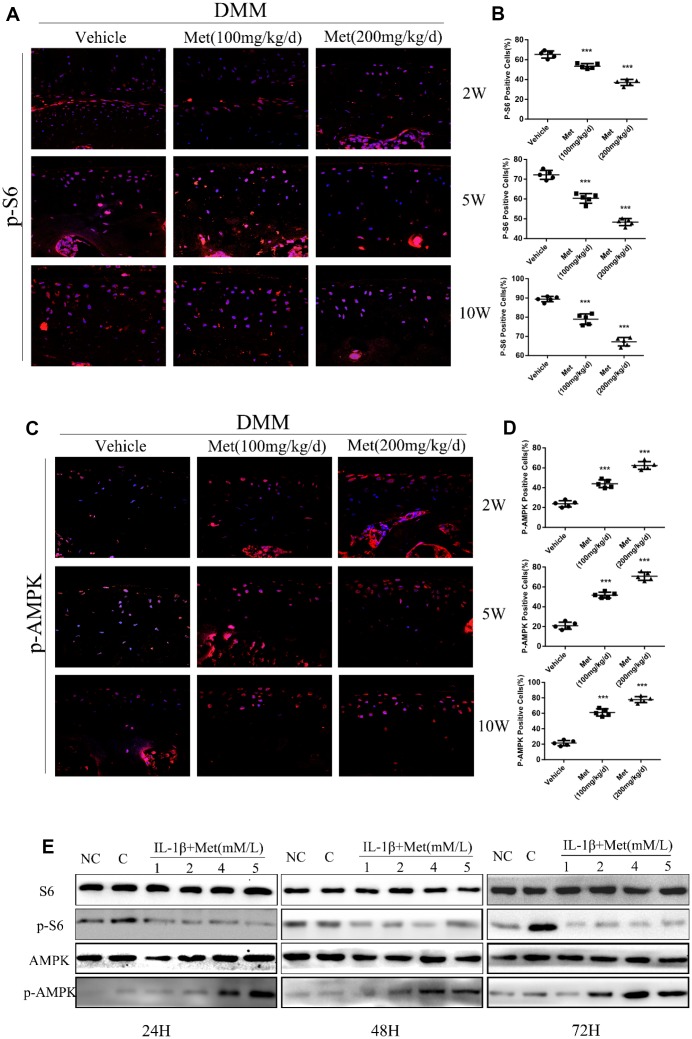
**Metformin reduces the expression of p-S6 protein and affects the expression of other related pathway proteins.** (**A**) Immunofluorescence detection of p-S6 in tibial cartilage at 2, 5, and 10 weeks after destabilization of the medial meniscus (DMM) surgery. (**B**) Quantitation of cells positively stained for p-S6. ^***^P < 0.001 between the two groups. (**C**) Immunofluorescence detection of p-AMPK in tibial cartilage at 2, 5, and 10 weeks after DMM surgery. (**D**) Quantitation of cells positively stained for p-AMPK. ^***^P < 0.001 between the two groups. And (**E**) the western blot analyses of the protein expression levels of selected proteins in primary chondrocytes which were induced with IL-1β (10 ng/mL) and co-cultured with metformin (1, 2, 4, and 5 mM) for 24 h, 48 h and 72 h. (NC = normal control, C = IL-1β).

The AMPK/mTORC1 signaling pathway participates in cartilage degeneration and chondrocytes aging. We therefore determined the expression of p-S6, phosphorylated S6 ribosomal protein, which can reflect the activation of mTORC1 in articular cartilage of control and experimental mice. Consistent with our previous results, p-S6 was highly expressed in the superficial layer of articular cartilage in DMM mice. However, ribosomal S6 protein phosphorylation was significantly inhibited in articular cartilage after treatment with metformin (Figure 4A, 4B). Moreover, metformin treatment resulted in a dose-dependent enhanced polarization of the AMPK signaling pathway, and inhibition of mTORC1 in cultured chondrocytes (Figure 4C). Besides, we also examined the expression of LC3, an autophagy marker, in each treatment group. The results showed that the metformin treatment group was able to increase the expression of LC3, suggesting that it had an effect of promoting autophagy (Supplementary Figure 5).

**Figure 5:**
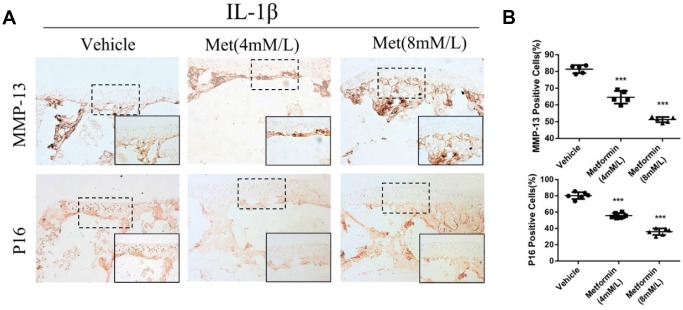
**The change of protein expression in cartilage explants.** (**A**) Immunohistochemical detection of MMP-13 and p16^INK4a^ in cartilage explants which were stimulated with IL-1β (50 ng/mL) for 48 h, and then co-cultured with metformin (4 mM and 8 mM) for 5 days. (**B**) Quantification of cells positively stained for matrix metalloproteinase-13 and p16^INK4a^. ^***^P < 0.001 between the two groups.

We further investigated the effect of metformin on AMPK/mTORC1 signaling in cartilage explants. Immunohistochemical staining showed that the AMPK signaling pathway was activated, but the mTORC1 signaling pathway was inhibited in metformin-treated cartilage explants. Simultaneously, the expression levels of p16^INK4a^, MMP-3 and MMP-13 decreased with increasing metformin concentrations (Figure 5A, 5B; Supplementary Figure 4A, 4B).

To further demonstrate the regulatory relationship between metformin and the MAPK/mTORC1 signaling pathway, we stimulated primary chondrocytes with an autologous agonist rapamycin and an autophagy inhibitor 3-ME. The results of western blots showed that the expression of p-S6 was decreased after treatment with rapamycin, and the treatment with rapamycin attenuated IL-1β-induced MMP-13 expression and increased Col2a1 expression, which was consistent with the metformin treatment group results. The 3-ME treatment results were the opposite (Supplementary Figure 6).

These results showed that metformin effectively alleviated cartilage degradation and aging by regulating the AMPK/mTOR signaling pathways.

## DISCUSSION

This study established the essential role of metformin in the pathogenesis and progression of OA. We propose a pathway in which metformin activates AMPK and suppresses mTORC1, to attenuate cartilage degradation and aging, and to alleviate the development of OA. Our findings showed a functional pathway important for OA development, and identified oral metformin administration as a potential therapy for OA.

The persistent degeneration of OA chondrocytes is the main cause of the exacerbation of joint symptoms, which is mainly caused by the degradation of the extracellular matrix. Metalloproteinases (MMPs) are the major enzymes involved in matrix degradation, and degrade one or more types of extracellular matrices. MMP-13 is the main enzyme for the degradation of type II collagen in the extracellular matrix of OA [39] and MMP-3 can regulate the activity of other MMPs and directly degrade type II collagen, which is involved in the progress of OA. Overall, inhibition of MMP-13 and MMP-3 effectively alleviated the degradation of COL2al, thereby maintaining the extracellular matrix structure of cartilage and reducing the progression of OA.

In order to illustrate the specific role of metformin in the body, we constructed an OA model to study the effects of the drug in animal models of OA and cartilage explants at different times and concentrations. Our results showed that the degree of degradation of cartilage matrix during the progression of OA was significantly improved, compared with the control group, with increased times and concentrations of metformin. Using immunohistochemical staining, we showed that the main enzymes that degrade the cartilage matrix, the expression level of MMP-13 and MMP-3 positive cells, gradually decreased with the increase of metformin concentrations. Similarly, under chondrocyte culture conditions, the protein expression and mRNA levels of MMP-13 and MMP-3 also decreased with time and concentration. At the same time, the protein expression level of cartilage matrix synthesis protein, COLII, and the mRNA expression level of SOX9 also increased with increases in metformin concentrations. Together, these results showed that metformin alleviated cartilage matrix degradation and stimulated cartilage matrix synthesis.

Recent studies by Diekman et al. [36] suggested that cellular senescence drives a functional decline of numerous tissues during aging, by limiting regenerative proliferation or by producing proinflammatory molecules known as the senescence-associated secretory phenotype (SASP), such as MMP-13 and IGF, which result in the degeneration of articular cartilage. Although the specific mechanism of cartilage aging and OA is unclear, it has been confirmed that the effect of chondrocyte aging on OA is more likely to result from excessive SASP [21, 32, 36]. In the present study, we demonstrated that the expression levels of P16^INK4a^ positive cells in chondrocytes showed a downward trend with increasing metformin concentrations in animal models and cartilage explants or in chondrocyte cultures. This indicates that metformin delays the secretion of SASP by delaying the aging of chondrocytes, thereby alleviating the effect of SASP on cartilage, and ultimately protects articular cartilage from degradation.

However, how metformin regulates the progression of OA is still unclear. In previous studies, many signaling pathways and related proteins have been reported to be involved in the pathological process of OA. Among them, the AMPK/mTOR signaling pathway, which has been recently studied, has been shown to be important.

AMPK is a highly conserved regulator of cellular energy metabolism that plays an important role in regulating cell growth, proliferation, survival, and regulating energy metabolism in the body [27, 40–41]. The mammalian target of rapamycin (mTOR) is an evolutionarily highly conserved atypical serine/threonine protein kinase belonging to the phosphatidylinositol kinase-associated kinase family. It mainly regulates cell growth, proliferation, apoptosis, and autophagy [42–44]. Under physiological and pathological conditions, the mTOR signaling pathway plays an important role in protein translation, synthesis, cell metabolism, and stress responses.

When the AMPK signaling pathway is activated, it promotes autophagy [45–47]. At the same time, phosphorylation of AMPK blocks the phosphorylation of the mTOR signaling pathway and inhibits its corresponding biological function [30, 48], ultimately regulating the progression of OA. Studies [49–51] have reported that reduced AMPK activity in chondrocytes is associated with OA and senescence. Metformin is therefore an activator of the AMPK signaling pathway, resulting in polarization of the AMPK signaling pathway and promotion of autophagy [45–46].

Immunofluorescence staining of our OA model showed that with the increase of metformin concentration, there was increased expression of p-AMPK positive cells, and increased expression of p-AMPK protein in chondrocytes, which showed that metformin treatment activated the AMPK signaling pathway in a dose-dependent manner. However, with an increase of metformin concentration, the expressions of p-S6 positive cells and p-S6 protein in chondrocytes decreased, indicating that the mTORC1 signaling pathway was significantly inhibited. We confirmed in animal experiments and cell experiments as well as cartilage explant experiments that metformin had a positive effect on the treatment of OA, both *in vivo* and *in vitro.*

To further verify that metformin delayed OA progression by regulating the AMPK/mTOR signaling pathway and autophagy levels, we examined the expression of LC3 in primary chondrocytes after metformin intervention. The results showed that metformin promoted the increase of autophagy. In addition, we demonstrated the regulation of the AMPK/mTOR signaling pathway by metformin by autophagy inhibitors and activators, and we showed that metformin can alleviate the increase of catabolism in OA and the decrease of type II collagen.

Metformin has been clinically used for many years. From its initial use in diabetes treatment to its current use as an anti-tumor, anti-inflammatory, and anti-aging treatment, it has shown a wide variety of uses in the clinic. In addition, the results of the present study broadens the potential clinical value of metformin [52–53]. We found that the pathological characteristics of the cartilage and cartilage matrix of OA in the animal DMM model effectively reduced the progression of OA, alleviated the degradation of the cartilage matrix, stimulated the expression of cartilage matrix-related protein, and promoted the synthesis and secretion of the cartilage matrix. It also played an important role in inhibiting aging of chondrocytes. By modulating the AMPK/mTOR signaling pathway, it is finally possible to alleviate the progression of OA. The results showed that metformin inhibited the degradation of the cartilage matrix by primary chondrocyte matrix metalloproteinase stimulated by IL-1β through the AMPK/mTORC1 signaling pathway, to reduce the destruction of articular cartilage in DMM mice, and to stabilize the cartilage matrix. These findings indicate that metformin is a potential target for the treatment of extracellular matrix degradation in OA chondrocytes.

## MATERIALS AND METHODS

### Animals

OA was induced in 8-week-old male C57BL/6 mice by destabilization of the medial meniscus (DMM) of the right knee, followed by random division into three groups [surgery group (DMM), DMM + Metformin group (100 mg/kg/d or 200 mg/kg/d; n = 24 per division). The day after surgery, the mice received metformin by oral gavage. We performed histological analysis using Safranin O-Fast Green staining and graded articular cartilage degeneration using the Osteoarthritis Research Society International (OARSI) guidelines.

### Treatment of primary articular chondrocytes and cartilage explants

Primary articular chondrocytes were isolated from newborn mice (within 3 days), induced with IL-1β (10 ng/mL) for 24 h, and then treated with metformin (1, 2, 4, and 5 mM). Cellular protein and mRNA were extracted after 24 h, 48 h, and 72 h. The quantitative real-time polymerase chain reaction (Q-PCR) and western blot analyses were conducted to detect relative protein expressions.

Cartilage explants were obtained from the tibial plateau cartilages of 8-week-old mice as previously described, and cultured in 10% fetal bovine serum in Dulbecco’s Modified Essential Medium /F-12 for 24 h, then stimulated with IL-1β (50 ng/mL) for 48 h. Finally, they were co-cultured with metformin (4 mm and 8 mm, n = 6 explants per condition) for 5 days.

Primary articular chondrocytes were induced with IL-1β (10 ng/mL) and treated with metformin (5 mM/L) or rapamycin (500 ng/mL) for 24 h. 3-ME(5 mM/L) was added after IL-1β (10 ng/mL) and co-cultured with metformin (5 mM/L) for 24 h. Protein was extracted after 24 h and the protein expression level was detected by western blotting.

### Antibodies

Rabbit monoclonal antibodies were: MMP-13, MMP-3, LC3 and COL2al; all, 1:100 (Abcam, Cambridge, UK); AMPK, p-AMPK, S6, p-S6; all, 1:100 (Cell Signaling Technology, Danvers, MA, USA); rabbit polyclonal antibody to p16^INK4a^ (Proteintech Group, Rosemont, IL, USA); rabbit/mouse secondary antibody; immunohistochemical and immunofluorescence staining concentration, 1:200; western blot concentration, 1:1,000 (Beijing Ray Antibody Biotech, Beijing, China).

### Western blotting

Cellular proteins were extracted from primary chondrocytes for immunoblot experiments. Protein extracts were electrophoresed on 8‒12% denaturing gels and transferred to a polyvinylidene fluoride membrane, followed by incubation in 5% skim milk at room temperature for 1.5 h, which was prepared in tris-buffered saline with tween 20 (TBST). After washing three times with TBST, the membranes were incubated with the corresponding antibodies for 12 h at 4°C. After washing three times, the corresponding secondary antibody was added and incubated for 1 h at room temperature, then washed three times, followed by color development.

### RNA extraction and quantitative real-time PCR (qPCR)

Total RNA was isolated by the RNeasy Plus Mini Kit (Qiagen, Hilden, Germany). To completely remove the gDNA, the dorsal horn samples were treated with a DNase (RNAqueous^®^-4PCR Kit, Ambion), followed by treatment with the inactivation reagent recommended by the manufacturer. Total RNA integrity and quantity were determined using an Agilent 2100 Bioanalyser (Agilent Technologies, San Jose, CA, USA). Only RNA with an A260/A280 ratio between 1.8 and 2.1 was used. Reverse transcription was conducted using an Omniscript RT Kit (Qiagen), according to manufacturer’s instructions. Finally, we used the LightCycler^®^ 96 system to analyze the mRNA expression levels in cells.

### Safranin O fast green staining

Paraffin sections containing intact articular sections were dewaxed and hydrated, stained with hematoxylin iron for 10 s, then rinsed with water for a few min, stained with 0.1% fast green aqueous solution for 5 min, immersed in glacial acetic acid for 5 s, and blown dry. After incubation in a 0.5% Safranin O dye solution for 5 min, the sections were dried at room temperature, made transparent with xylene treatment for 5 min, and finally sealed with a neutral gel.

### Immunohistochemical and immunofluorescence staining

The paraffin-embedded tissue was sectioned in a sodium hydroxide buffer at 65°C in a constant temperature water bath for about 14 h to renature the antigen. Next, the endogenous peroxidase was quenched with 3% hydrogen peroxide for 10 min, washed three times with phosphate-buffered saline, added to goat serum for 1 h at room temperature, followed by dropwise addition of the corresponding primary antibody at 4°C. After 14‒16 h, after washing three times with phosphate-buffered saline, the sections were incubated with biotinylated secondary antibody (anti-rabbit immunoglobulin) at room temperature for about 1 h, and finally developed in the dark and sliced. After counterstaining with hematoxylin, the tablets were mounted.

## Supplementary Material

Supplementary Figures
