# Supplementary material for: Metformin attenuates cartilage degeneration in an experimental osteoarthritis model by regulating AMPK/mTOR
Source: Aging (Albany NY). 2020 Jan 16;12(2):1087–103. doi: 10.18632/aging.102635 (PMC7053618; doi:10.18632/aging.102635)
Supplement: Supplementary Figures [file aging-12-102635-s001..pdf]

SUPPLEMENTARY FIGURES

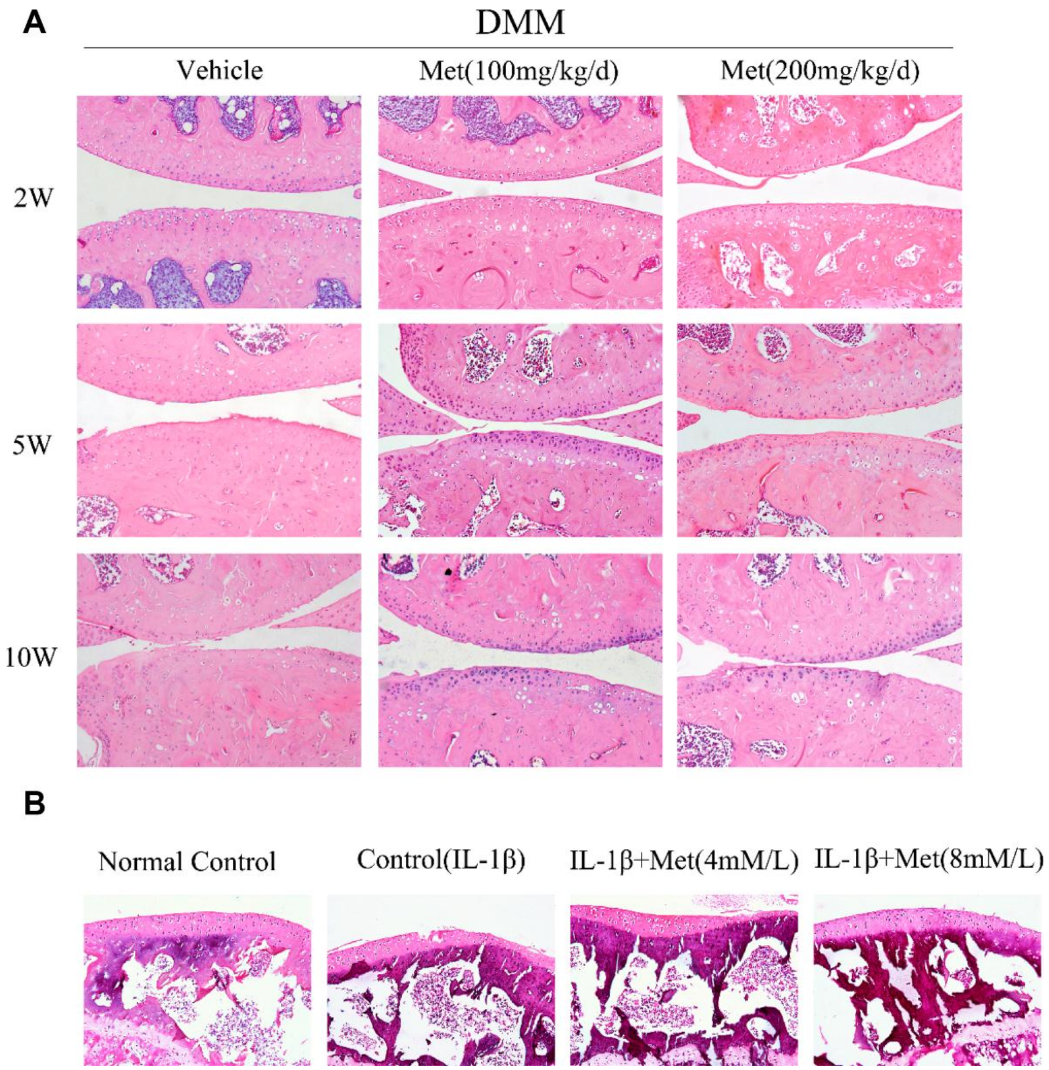

Supplementary Figure 1. HE staining of the destabilization of the medial meniscus (DMM), DMM + Metformin joints and cartilage explants. (**A**) HE staining of paraffin section of mouse DMM and DMM+metformin joints. (**B**) HE staining of cartilage explants treated with IL-1β and metformin (4 mM and 8 mM) for 5 days.

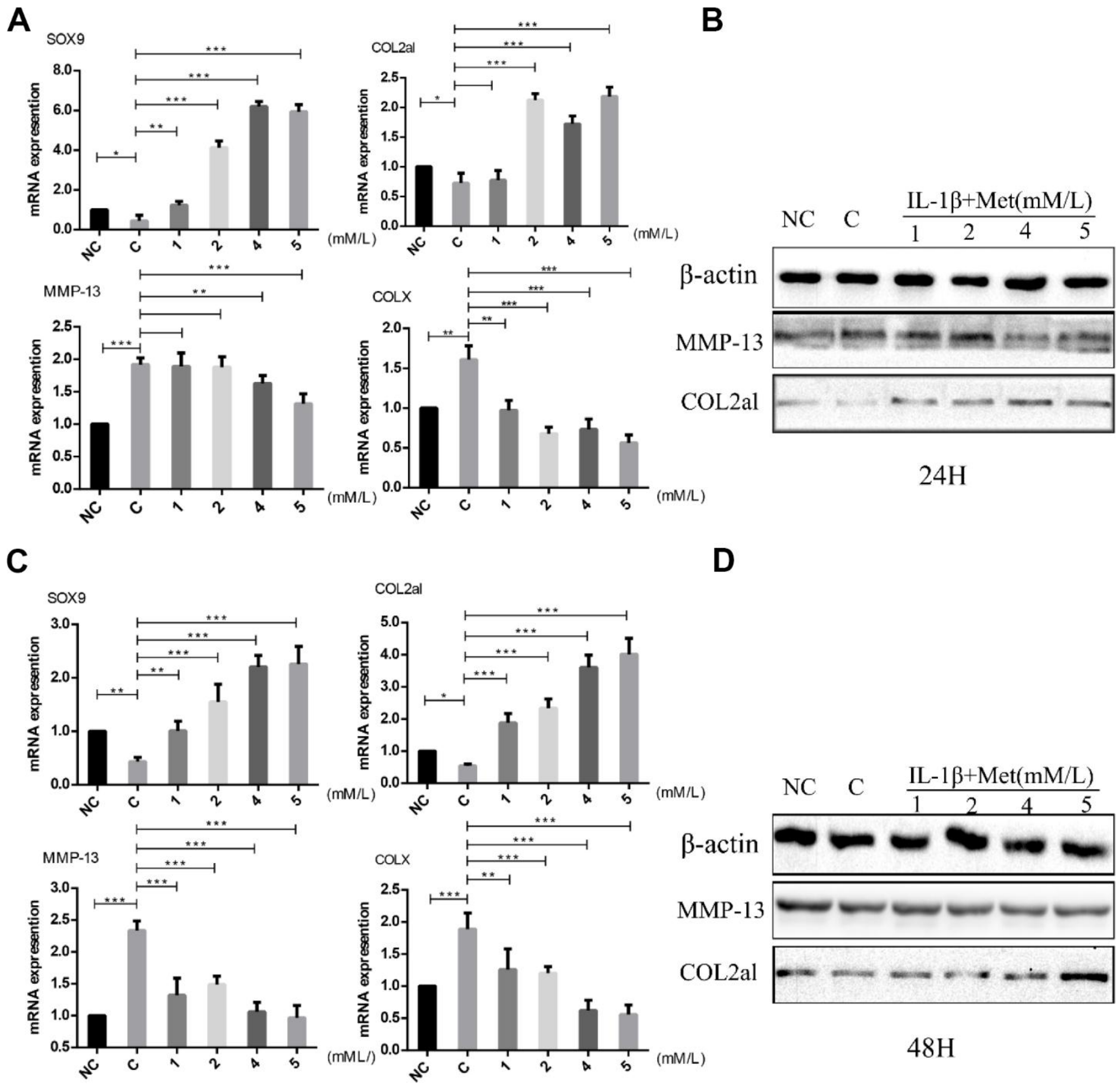

Supplementary Figure 2. Metformin reduces the degradation of cartilage matrix. (**A**, **C**) The mRNA expression levels of MMP-13, SOX9, COLX and COL2al, and (**B**, **D**) the western blot analyses of MMP-13 and COL2al. Primary chondrocytes were induced with IL-1β and then co-cultured with metformin (1, 2, 4, and 5 mM) for 24 and 48 h.

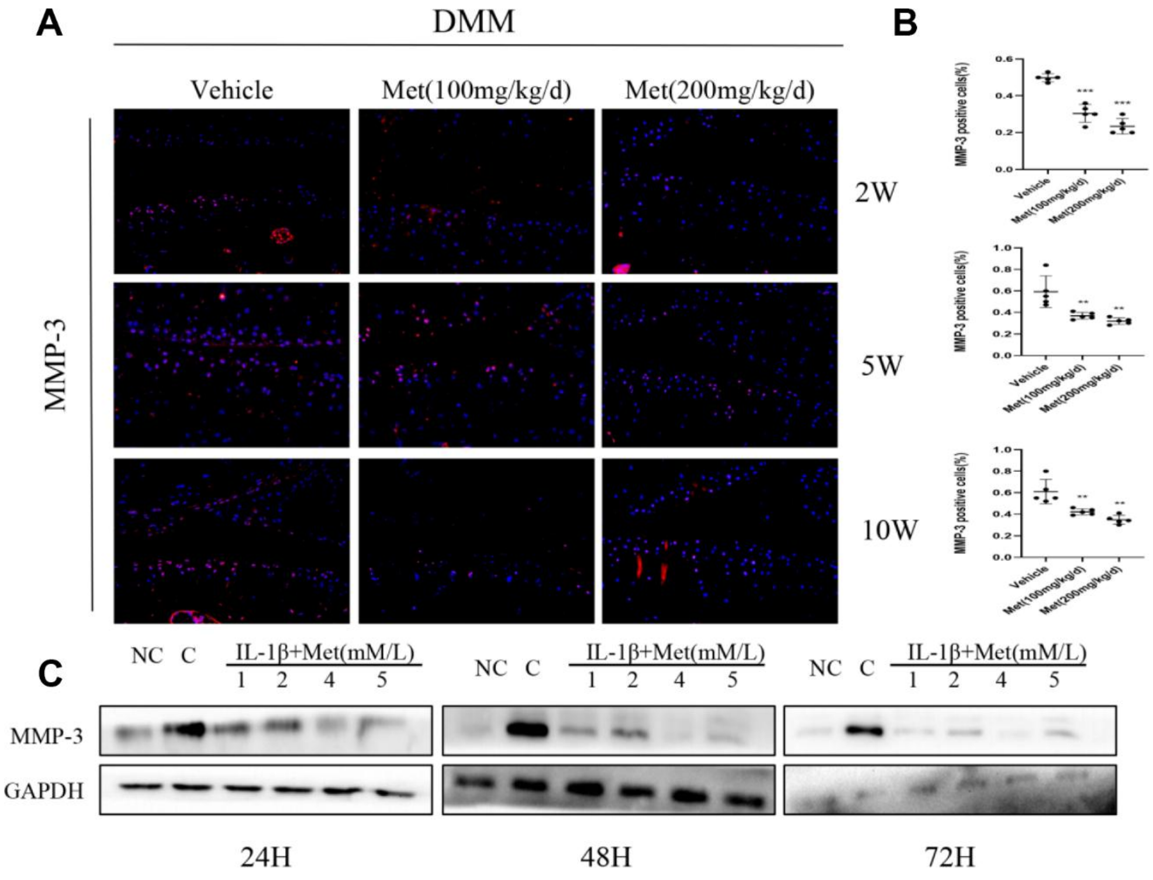

Supplementary Figure 3. Metformin reduces the expression of MMP-3 in the medial meniscus (DMM) and DMM + Metformin joints. (**A**) Immunohistochemical detection of MMP-3 in tibial cartilage at 2, 5, and 10 weeks after destabilization of the medial meniscus surgery. (**B**) Quantification of cells positively stained for matrix metalloproteinase-3 (MMP-3). $^{***}P < 0.001$ between the two groups. (**C**) Western blot analyses of MMP-3. Protein extracted from the primary chondrocytes which were stimulated with IL-1β and co-cultured with metformin (1, 2, 4, and 5 mM).

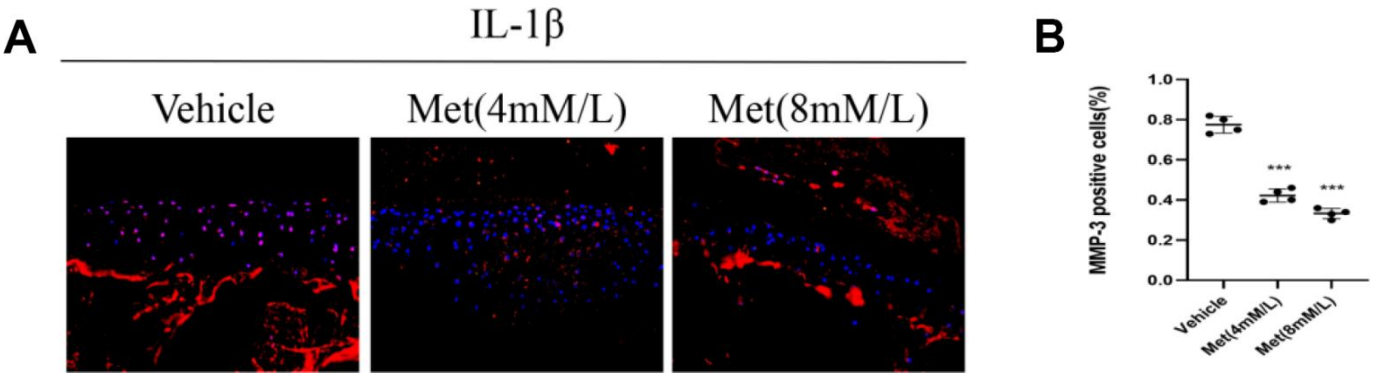

Supplementary Figure 4. Metformin reduces the expression of MMP-3 in the cartilage explants. (**A**) Immunohistochemical detection of MMP-3 in cartilage explants which were stimulated with IL-1β (50 ng/mL) and then co-cultured with metformin (4 mM and 8 mM). (**B**) Quantification of cells positively stained for matrix metalloproteinase-3. $^{***}P < 0.001$ between the two groups.

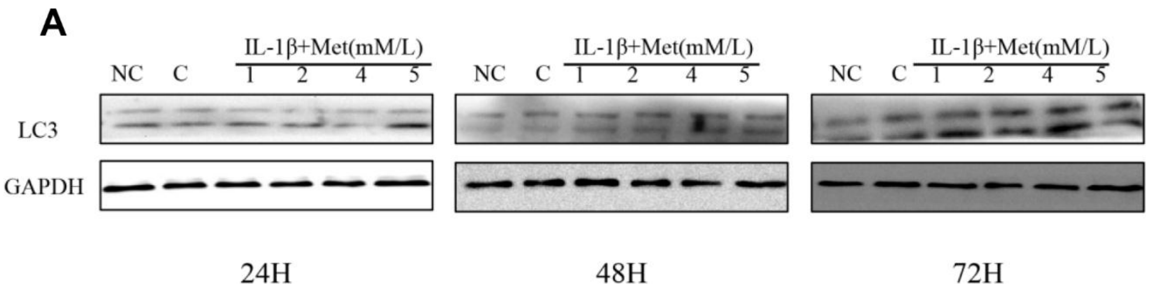

Supplementary Figure 5. The expression level of the autophagy marker LC3. (**A**) Western blot analyses of LC3. The results showed that metformin promoted the increase of LC3 II/I.

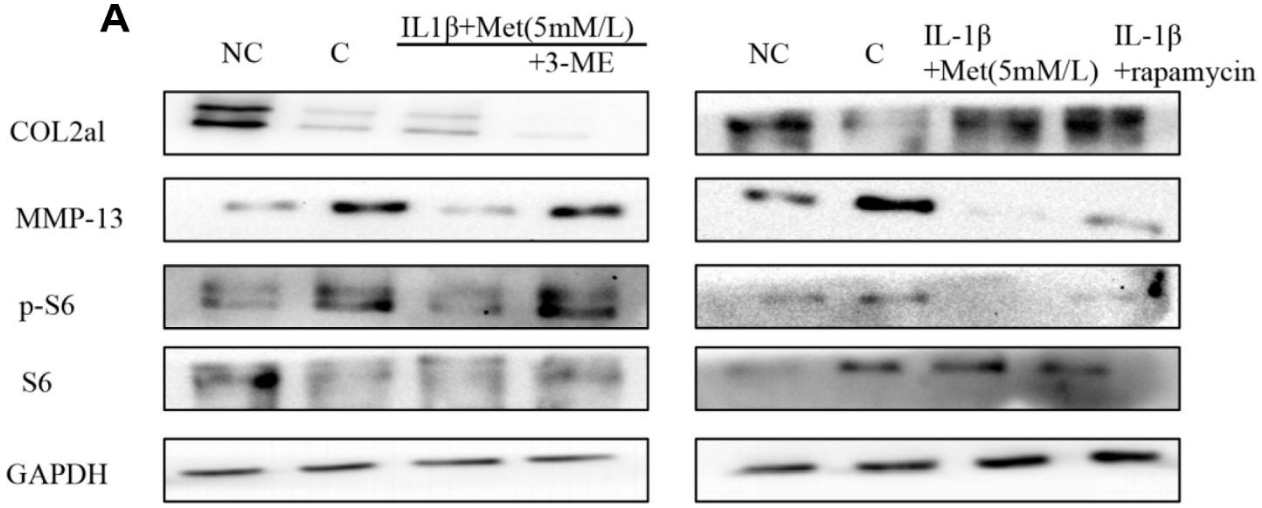

Supplementary Figure 6. The change in protein expression after the stimulation of an autologous agonist rapamycin and an autophagy inhibitor 3-ME. (**A**) Western blot analyses of COL2al, MMP-13 and p-S6. Primary chondrocytes were induced with IL-1β (10 ng/mL) and treated with metformin (5 mM/L) or rapamycin (500 ng/mL). The 3-ME (5 mM/L) was co-cultured with metformin after IL-1β stimulation. The expression of p-S6 was decreased after treatment with rapamycin (500 ng/mL), and the treatment with rapamycin attenuated IL-1β-induced MMP-13 expression and increased Col2a1 expression, which was consistent with the metformin treatment group results. The 3-ME (5mM/L) treatment results were the opposite.
